# Dysregulated iron metabolism in polycythemia vera: etiology and consequences

**DOI:** 10.1038/s41375-018-0207-9

**Published:** 2018-07-24

**Authors:** Yelena Z. Ginzburg, Maria Feola, Eran Zimran, Judit Varkonyi, Tomas Ganz, Ronald Hoffman

**Affiliations:** 1 Division of Hematology Oncology, Icahn School of Medicine at Mount Sinai, New York, NY USA; 2 Third Department of Internal Medicine, Semmelweis University, Budapest, Hungary; 3 David Geffen School of Medicine, UCLA, Los Angeles, CA USA

## Abstract

Polycythemia vera (PV) is a chronic myeloproliferative neoplasm. Virtually all PV patients are iron deficient at presentation and/or during the course of their disease. The co-existence of iron deficiency and polycythemia presents a physiological disconnect. Hepcidin, the master regulator of iron metabolism, is regulated by circulating iron levels, erythroblast secretion of erythroferrone, and inflammation. Both decreased circulating iron and increased erythroferrone levels, which occur as a consequence of erythroid hyperplasia in PV, are anticipated to suppress hepcidin and enable recovery from iron deficiency. Inflammation which accompanies PV is likely to counteract hepcidin suppression, but the relatively low serum ferritin levels observed suggest that inflammation is not a major contributor to the dysregulated iron metabolism. Furthermore, potential defects in iron absorption, aberrant hypoxia sensing and signaling, and frequency of bleeding to account for iron deficiency in PV patients have not been fully elucidated. Insufficiently suppressed hepcidin given the degree of iron deficiency in PV patients strongly suggests that disordered iron metabolism is an important component of the pathobiology of PV. Normalization of hematocrit levels using therapeutic phlebotomy is the most common approach for reducing the incidence of thrombotic complications, a therapy which exacerbates iron deficiency, contributing to a variety of non-hematological symptoms. The use of cytoreductive therapy in high-risk PV patients frequently works more effectively to reverse PV-associated symptoms in iron-deficient relative to iron-replete patients. Lastly, differences in iron-related parameters between PV patients and mice with *JAK2 V617F* and JAK2 *exon 12* mutations suggest that specific regions in JAK2 may influence iron metabolism by nuanced changes of erythropoietin receptor signaling. In this review, we comprehensively discuss the clinical consequences of iron deficiency in PV, provide a framework for understanding the potential dysregulation of iron metabolism, and present a rationale for additional therapeutic options for iron-deficient PV patients.

## Introduction

Polycythemia vera (PV), essential thrombocytosis (ET), and primary myelofibrosis (MF) are the classical BCR-ABL negative myeloproliferative neoplasms (MPNs). These blood cancers represent a heterogeneous group of clonal hematopoietic stem cell disorders with constitutively activated physiologic signal-transduction pathways [1]. PV is characterized by erythrocytosis, bone marrow erythroid and megakaryocytic hyperplasia, fatigue, aquagenic pruritus, microvascular symptoms, and symptomatic splenomegaly. Complications of PV include a significantly increased risk of arterial and venous thrombosis (41%) and the potential for evolution to MF (10–20%) and MPN-blast phase (3–10%), significantly reducing survival [2–4]. Most patients with PV present with iron deficiency at diagnosis [5, 6], even prior to the onset of therapeutic phlebotomy, the mainstay of treatment. Iron deficiency is often exacerbated by repeated phlebotomies, a desired effect thought to limit the accelerated erythropoiesis by restricting a necessary component of hemoglobin synthesis. This can lead to symptoms such as cognitive impairment and fatigue, even in the absence of anemia [7–10]. Recent studies demonstrate that available therapies might improve PV-related symptoms in part by reversing iron deficiency [11]. The genesis of iron deficiency in PV and its effect on disease manifestations and natural history have been explored to a limited extent. In this review, we focus on what is known about iron metabolism and erythropoiesis in PV. In addition, we build on recent advances in understanding the regulation of iron metabolism to enhance our understanding of PV pathophysiology and ultimately propose alternative therapeutic options.

## Underlying mutations in polycythemia vera

**Fig. 1:**
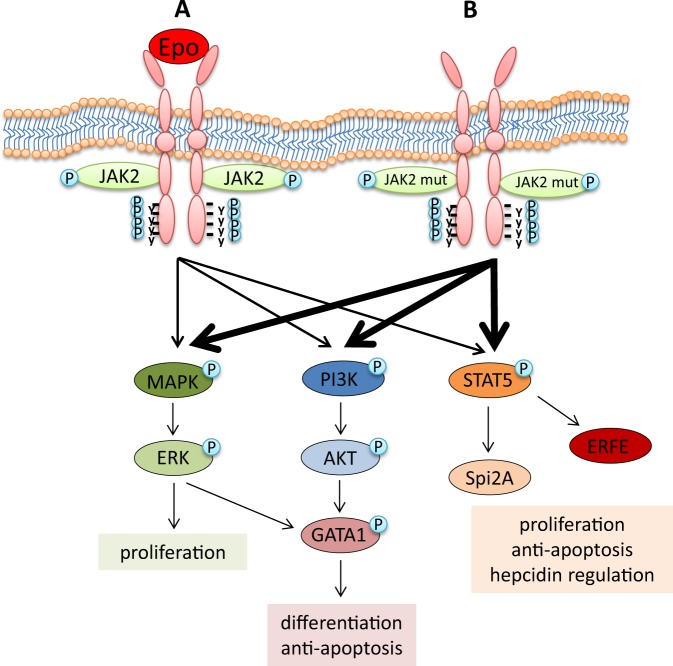
JAK2 signaling. **a** Binding of Epo to EpoR results in receptor homodimerization and autophosphorylation (marked with P) of JAK2, which in turn mediates the phosphorylation of key tyrosine residues of EpoR, docking sites for downstream effectors, including STAT5, PI3K, and MAPK, and influencing downstream pathways involved in proliferation, differentiation, preventing apoptosis, and regulating iron metabolism. **b** In polycythemia vera, JAK2 mutations result in a constitutively active signaling in the absence of Epo. Epo erythropoietin, JAK2 janus kinase 2, JAK2 mut JAK2 mutation (i.e., V617F and exon 12), STAT5 signal transducer and activator of transcription 5, PI3K phosphatidylinositol 3 kinase, MAPK mitogen-activated protein kinase, ERK extracellular signal–regulated kinase, AKT serine/threonine-protein kinase B, GATA1 globin transcription factor 1, Spi2A spindlin family member 2A, ERFE erythroferrone

The genetic basis of PV was largely speculative until the discovery of driver mutations involving janus kinase 2 (JAK2). JAK2 is a non-receptor tyrosine kinase that transduces erythropoietin receptor (EpoR) (as well as granulocyte-colony stimulating factor and thrombopoietin  receptors) signaling. Activation of JAK2 triggers multiple signaling pathways regulating erythroid precursor survival, proliferation, and differentiation (Fig. 1). The most common JAK2 driver mutation is *JAK2 V617F* which results in constitutive erythropoietin (Epo)-independent JAK/STAT (signal transducer and activator of transcription) signaling and upregulation of genes downstream of the JAK/STAT pathway [12–17]. The vast majority (approximately 96%) of PV patients are *JAK2 V617F* positive. An additional 2–3% harbor mutations in exon 12 of *JAK2* [18, 19], and the remainder have rare mutations in LNK (SH2B3) or other yet to be determined mutations in negative regulators of JAK/STAT signaling [20]. Among patients with MPNs, mutations in exon 12 of *JAK2* are found only in PV. These patients exhibit significantly higher hemoglobin levels and typically present with isolated erythrocytosis [21, 22], suggesting that different regions of the *JAK2* gene may be associated with different degrees of lineage restriction. *JAK2 V617F* is found in approximately 50% of ET and PMF patients, but *JAK2 V617F* allele burden is usually higher in PV than ET patients [23, 24] and *JAK2 V617F-*positive ET patients generally have higher hemoglobin and lower platelet counts as compared to *JAK2 V617F*-negative ET patients [25, 26]. These findings confirm that *JAK2 V617F* results in a more PV-like picture among ET patients but raise the question of how a single mutation can result in phenotypic diversity among the MPNs.

## Current treatment approaches for polycythemia vera patients

The primary goals of treating PV patients are to ameliorate symptoms, reduce the risk of thrombosis, and prevent transformation to MF and/or MPN-blast phase. Aspirin is universally administered to all eligible PV patients without a contraindication as thrombosis prophylaxis based on evidence from Italian investigators who demonstrated a strong trend toward decreased morbidity and mortality from cardiovascular causes in aspirin-treated PV patients [27]. Furthermore, normalization of the hematocrit (HCT) levels of PV patients correlates with reduced risk of thrombosis and represents the primary therapeutic goal given the frequency of thrombotic complications and the high consequent morbidity and mortality [28, 29]. This can be achieved by therapeutic phlebotomy alone or in combination with cytoreductive agents, including hydroxyurea, interferon alpha, or busulfan. Recently, the JAK1/2 inhibitor ruxolitinib (i.e., Jakafi) was approved for PV patients intolerant of or refractory to hydroxyurea [28–30]. Although the majority of patients exhibit clinical responses to ruxolitinib (e.g., HCT normalization, alleviation of systemic symptoms, and reduction of splenomegaly), ruxolitinib therapy, like treatment with other cytoreductive agents, has not been shown to alter the natural history of the disease. As a consequence, patients who are <60 years of age with no history of thrombosis (low-risk PV) are maintained on therapeutic phlebotomy alone, often for many years.

The beneficial effect of phlebotomy in PV may depend not only on removing excess red blood cells (RBCs) to reduce the risk of thrombosis, but also on the resulting iron deficiency that inhibits erythropoiesis. Iron restriction decreases hemoglobin synthesis, and thereby decreases mean corpuscular volume (MCV) which in part alone accounts for the decreased HCT after phlebotomy. However, iron deficiency also regulates the proliferation and differentiation of erythroblasts by a mechanism not yet fully elucidated. What we do know is that the “iron restriction response” employs multiple iron-sensitive molecular pathways to inhibit the differentiation and proliferation of erythroblasts and cause anemia in iron-deficient states [31–34]. The proposed mechanisms involve mitochondrial aconitase enzymes [31], transferrin receptor 2 (TfR2) [32, 33], and scribble-mediated EpoR regulation [34], as well as effects on the erythroblast cell cycle [35]. It is possible that the disease process causes erythroblasts in PV to be less sensitive to iron deficiency, allowing them to differentiate and proliferate despite iron restriction.

In total, the target HCT goal of <45% is associated with a decrease in cardiovascular complications [36, 37]. However, controlling HCT has not been clearly shown to decrease symptom burden or the risk of disease progression [38]. In fact, repeated phlebotomy may exacerbate iron deficiency-related symptoms and has been associated with worsening of disease-related symptoms such as pruritus [39]. Furthermore, a high phlebotomy requirement (>4 per year) is associated with increased risk of thrombosis [40], but whether this is due to worsening iron deficiency, other consequences of phlebotomy, or to the underlying disease remains poorly understood. High phlebotomy requirements often indicate the need for initiating cytoreductive therapy which may reverse symptoms of iron deficiency possibly by decreasing the frequency of phlebotomy.

## Clinical manifestations of iron deficiency in the absence of anemia

Iron deficiency is defined by insufficient iron availability to meet physiological needs in the presence or absence of anemia. Because >60% of the body’s iron is found within hemoglobin, and anemia is the most easily recognizable sign of iron deficiency, the terms “iron deficiency” and “iron deficiency anemia” have inaccurately become synonymous. The dissociation of anemia from iron deficiency is particularly striking in PV, but there are also other situations where iron availability for erythropoiesis is sufficient to prevent anemia but insufficient to avoid other consequences of iron deficiency. Recently, deleterious effects of non-anemic iron deficiency on educational achievement in children [41], reduced birthweight during pregnancy [42], cognitive impairment and fatigue in women [43, 44], and decline in physical performance [43, 44, 10] have been documented and demonstrated to be reversible with oral iron supplementation [43, 44, 8, 10]. Non-anemic iron deficiency has also been linked to decreased muscle function [45, 46] and impaired neonatal brain development, possibly leading to long-term neurological impairments, including deficits in learning and memory in experimental animals [47]. Although individual studies are not always in agreement about the specific clinical consequences of iron deficiency, fatigue and its reversibility with iron supplementation has been documented in studies of animals and humans [10].

Fatigue as a general symptom of non-anemic iron deficiency may be related to the ubiquitous requirement for iron in all cells, particularly for the formation of heme as part of myoglobin, cytochromes, and nitric oxide synthase as well as in multiple enzymes required for the generation of adenosine triphosphate [48–51]. Myoglobin is the oxygen storage protein in high-oxygen-requiring tissues, i.e., skeletal and cardiac muscle. In fact, iron repletion in non-anemic iron-deficient patients with congestive heart failure resulted in improved function, exercise tolerance, and quality of life [52–55] with evidence from a meta-analysis study of significant reduction in recurrent hospitalizations, cardiovascular mortality, and all-cause mortality [55]. Furthermore, iron is critical for energy metabolism due to its contribution to (1) heme-containing enzymes such as cytochrome *c* (in the electron transport chain) and cytochrome *c* oxidase (in the oxidative phosphorylation pathway) as well as (2) non-heme-containing enzymes including NADH dehydrogenase and succinate dehydrogenase (in the respiratory chain) and aconitase (in the Kreb’s cycle) [48]. Lastly, iron is a necessary component of key proteins involved in DNA replication, repair, and cell cycle progression [51].

## Iron metabolism and its regulation

Since most of the body iron is destined for hemoglobin synthesis, erythropoiesis and iron metabolism are inextricably linked. Iron must be tightly regulated to avoid shortfalls or excesses, which typically result in anemia or iron overload, respectively. The stable concentration of circulating iron, which enables erythropoiesis and other iron-requiring physiological processes, is maintained by baseline dietary absorption, storage, and recycling of iron. Recycled iron within bone marrow, spleen, and liver macrophages is either stored in cytosolic ferritin or exported to carrier proteins. Transferrin is the main iron carrier in circulation, and transferrin saturation of iron-binding sites, calculated as a ratio of serum iron to total transferrin iron-binding capacity, is approximately 20–45% under normal conditions. Iron deficiency is typically diagnosed on the basis of decreased transferrin saturation (i.e., <15%) and serum ferritin (<22 ng/mL), tests that effectively supplanted the historical gold standard for diagnosis, the absence of iron stores in bone marrow aspirate samples. Despite their ease of use, there are limitations to the sensitivity and specificity of transferrin saturation and serum ferritin in the diagnosis of iron deficiency [56–60]. First, there is a lack of established cutoffs to confidently distinguish “iron depletion” from “absolute iron deficiency”. Second, additional markers, such as serum soluble TfR1 (sTfR1) [61] or zinc protoporphyrin IX [62, 63], are in use to identify “iron restricted erythropoiesis”. When sTfR1 is combined with serum ferritin (i.e., log(sTfR1/ferritin)) using internal cutoffs, corresponding with 95th or 97.5th percentile distribution within a study population, it is highly sensitive in identifying clinically relevant iron restricted erythropoiesis [56, 57, 64], but because sTfR1 and log(sTfR1/ferritin) are not standardized, its clinical utility is limited.

**Fig. 2:**
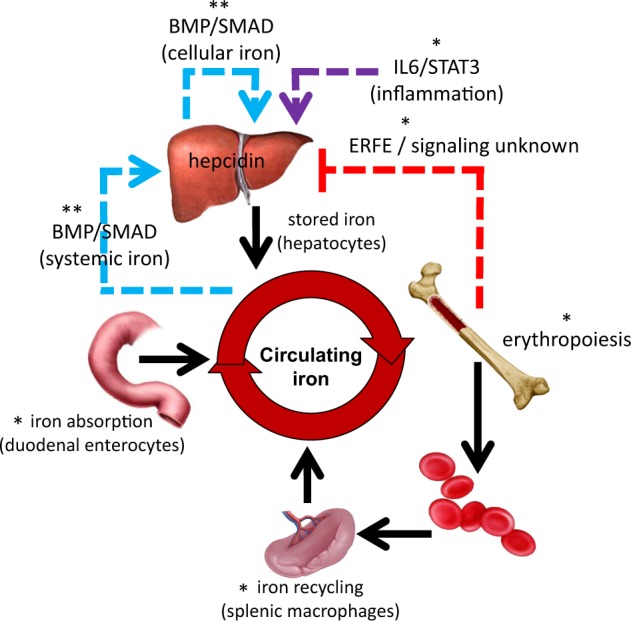
Central role of hepcidin in regulation of iron metabolism. Hepcidin regulates iron absorption, iron recycling, and iron released from stores. Hepcidin expression is enhanced by iron regulatory pathway (blue arrow) and inflammatory cytokines (violet arrow) and suppressed by erythropoiesis (red arrow). *Enhanced in polycythemia vera, **suppressed in polycythemia vera, BMP bone morphogenic protein, IL-6 interleukin-6, STAT3 signal transducer and activator of transcription 3

The peptide hormone hepcidin [65, 66], secreted primarily by hepatocytes, is the principal regulator of multiple events that lead to iron homeostasis [67], including dietary iron absorption, iron recycling by macrophages, and the release of iron from hepatic stores (Fig. 2). Hepcidin down-regulates iron release into the plasma by binding to and functionally down-regulating ferroportin [68, 69], the sole iron exporter [69]. Ferroportin expression in macrophages is also transcriptionally regulated by heme [70] and is under translational control by iron [71]. Hepcidin transcription is increased by iron loading [65] and by elevated levels of inflammatory cytokines [72] but is suppressed by tissue hypoxia [73], iron deficiency, and ineffective erythropoiesis.

The bone morphogenic protein (BMP) pathway plays a critical role in the iron-dependent regulation of hepcidin expression [74, 75]. The ligands BMP6 and BMP2, and the co-receptor hemojuvelin (HJV), stimulate BMP type I receptor (ALK 2/3) in iron overload states [75]. The holotransferrin-sensing complex of TfR1 with the hemochromatosis gene product, HFE, together with TfR2, interact with BMP receptor complex to modulate the BMP/SMAD1/5/8 signaling pathway and hepcidin transcription [75, 76]. The increase in hepcidin levels during inflammatory stress is primarily mediated by interleukin-6 (IL-6) [77, 78] and signaling via the STAT3 pathway in hepatocytes which results in iron sequestration within iron-recycling macrophages, hypoferremia, and restricted iron availability for erythropoiesis [78, 79] (Fig. 2).

Hepcidin suppression as a consequence of increased erythropoietic activity, irrespective of the degree of anemia, hypoxia, or Epo levels, has been repeatedly postulated to explain iron overload in diseases of ineffective erythropoiesis [80–83]. Numerous investigators have hypothesized that an “erythroid factor”, secreted by bone marrow erythroid precursors, functions as a hormone which suppresses hepcidin expression in the liver (Fig. 2). Several candidate erythroid-derived hepcidin regulators have been proposed. For example, circulating growth differentiation factor 15 (GDF15) levels are increased in patients with some diseases of ineffective erythropoiesis and GDF15 levels have been shown to correlate with low hepcidin levels [84]. However, studies in two conditions where hepcidin is suppressed during exuberant marrow hyperplasia, phlebotomized mice [85] and patients with myelodysplastic syndrome [86], demonstrate a poor correlation between GDF15 and hepcidin levels. More recently, a potential physiological regulator of hepcidin, erythroferrone (ERFE), was identified [87]. The role of ERFE was supported by the failure of hepcidin levels to be suppressed after phlebotomy in ERFE knockout mice, increased ERFE in mouse models of β-thalassemia, and increased hepcidin expression and decreased iron overload in β-thalassemic/ERFE knockout relative to β-thalassemic mice [87]. In addition, in β-thalassemia patients, serum ERFE concentrations are increased compared to healthy controls, inversely correlated with hepcidin concentrations, and decreased after RBC transfusion therapy [88]. Lastly, ERFE levels increase in normal blood donors in the days following blood donation and return to normal levels at the time donors are again eligible for donation [88]. ERFE has therefore emerged as an important physiological and pathological suppressor of hepcidin produced by erythroid precursors.

**Fig. 3:**
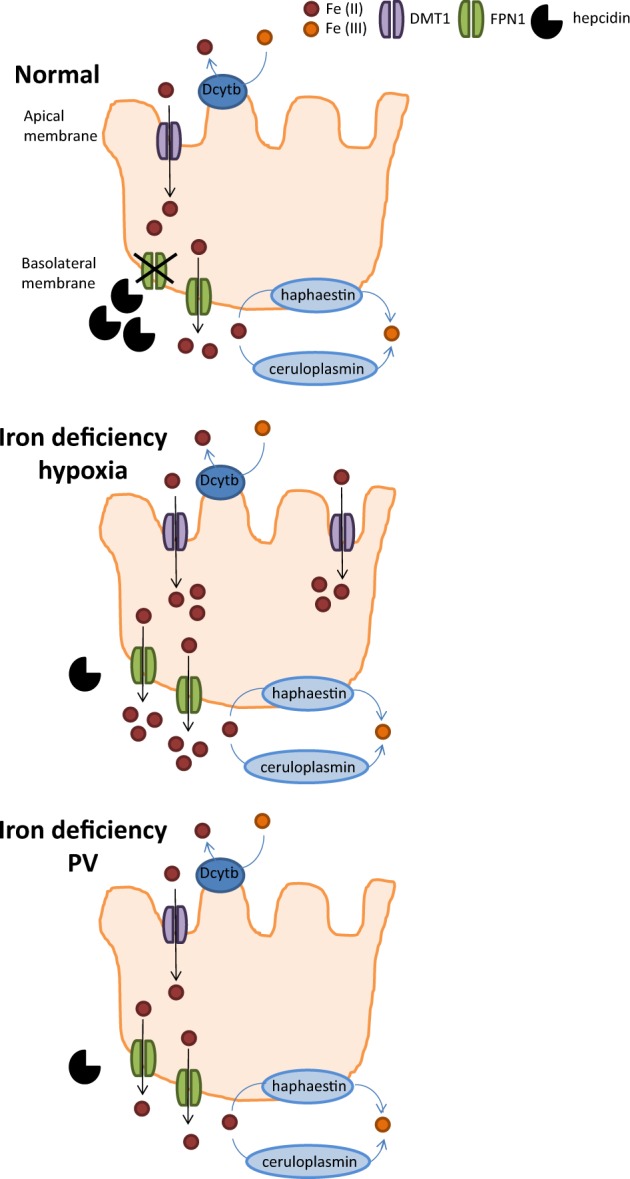
Regulating iron absorption by hepcidin-dependent and independent mechanisms. At the duodenal enterocyte, where iron is absorbed, hepcidin functions as a negative regulator at the basolateral surface, where ferroportin exports iron from duodenal enterocytes into the circulation. Hypoxia functions as a positive regulator at the apical surface where DMTI imports iron into duodenal enterocytes. Together, the presence of hypoxia in the gastrointestinal tract with suppression of hepcidin would provide the most potent stimulus for iron absorption. In PV, it is thus possible that, despite hepcidin suppression, hypoxia is diminished by erythrocytosis, preventing recovery from iron deficiency. DMTI divalent metal transporter 1, FPN ferroportin, PV polycythemia vera

Finally, hypoxia also regulates hepcidin in both an Epo-dependent and Epo-independent manner. Physiological responses to hypoxia are regulated by transcription factors known as hypoxia-inducible factors (HIFs). HIFs bind hypoxia-response elements to regulate genes central to erythropoiesis (e.g., Epo), angiogenesis, and metabolism. HIFs are degraded in the proteasome as a consequence of HIF hydroxylation by propyl hydroxylases in normoxic conditions. Propyl hydroxylase function requires iron and inactivating mutations in propyl hydroxylases result in increased HIF concentration and induction of erythrocytosis, overriding the upregulation of hepcidin associated with inflammation [89], resulting in hepcidin suppression [90]. Several in vitro studies suggest direct hepcidin regulation by HIFs [89, 91]. However, subsequent in vivo studies identified Epo-dependent pathways (e.g, via ERFE) as critical for HIF-induced hepcidin suppression [92, 93]. In addition, hypoxia induces iron absorption directly, in part independently of regulation by hepcidin and ferroportin. The typically hypoxic environment with consequently increased HIF2α in the small intestine induce iron uptake at the apical side of enterocytes and enhance iron absorption at the basolateral side (via ferroportin) independently of hepcidin [94] (Fig. 3). Lastly, recent in vivo data indicate that iron restriction enhances hypoxia responsiveness [95] by regulating HIF2α [96]. Taken together, iron metabolism in PV may be altered in light of the potential for concurrent involvement of aberrant erythropoiesis, an inflammatory milieu, decreased systemic iron concentration, and potentially altered hypoxia responsiveness directly influencing iron absorption in the small intestine.

## Iron metabolism in polycythemia vera

Because anemia is the best recognized indicator of iron deficiency, the diagnosis of iron deficiency in the context of PV, and thus without anemia, requires the identification of ferritin, transferrin saturation, and MCV below the lower limit of normal. In addition, symptoms associated with iron deficiency, including fatigue, global weakness, and neuromuscular disturbances, may overlap with those of PV such that attributing symptoms to a single underlying cause may be challenging. Furthermore, glossitis, cheilosis, koilonychias, restless leg syndrome, and Pica have all been reported in PV patients, and support the diagnosis of iron deficiency in this context. Decisions regarding the management of iron deficiency in PV are complex. On one hand, it is compelling to attempt symptom relief with iron supplementation and, on the other hand, attaining iron deficiency is an intended consequence of therapeutic phlebotomy which limits the degree of erythrocytosis. Although symptoms of iron deficiency rarely result in discontinuing therapeutic phlebotomy, initiating cytoreductive therapy may be warranted to enable HCT control with less intensive phlebotomy especially since iron supplementation often leads to worsening of erythrocytosis, requiring an increase in the frequency of phlebotomies and/or the institution of cytoreductive therapy.

Cytoreductive therapy may be associated with correction of the iron deficiency in PV patients. Whether this effect is achieved by decreasing phlebotomy requirement or by directly influencing regulators of iron metabolism is incompletely understood. For example, a recent phase III clinical trial in PV patients demonstrated significant reductions in HCT levels, splenomegaly, and PV-related symptoms in the ruxolitinib-treated group relative to those receiving best available therapy [11]. Notably, ruxolitinib treatment resulted in normalization of standard iron-related parameters in PV patients with baseline iron deficiency and hepcidin increased to a greater extent in ruxolitinib-treated PV patients relative to those treated with best available therapy. However, analysis of HepG2 cells treated with ruxolitinib in vitro led to decreased hepcidin expression at high concentration mainly by decreasing signaling via STAT3 [97]. These findings suggest that *enhanced* hepcidin expression in ruxolitinib-treated PV patients may be due to suppression of erythropoiesis and thus a presumed reduction in ERFE levels. It is conceivable that reversal of PV-related symptoms in ruxolitinib-treated patients is a consequence solely of suppressing clonal and/or normal erythropoiesis. However, it is also possible that, because iron deficiency- and PV-related symptoms often overlap, ruxolitinib-treated patients improve symptomatically due to the consequent decreasing in phlebotomy requirements, reversing iron deficiency in general, or specifically increasing hepcidin levels, resulting in iron sequestration without exacerbating systemic iron deficiency. For example, administration of exogenous hepcidin has been shown to reverse erythrocytosis and splenomegaly in *JAK2 V617F* mice [98]; the mechanism of action is likely sequestration of iron in splenic macrophages, inducing iron-restricted erythropoiesis.

In addition, analysis of bone marrow samples from *JAK2 V617F* and *exon 12* mutant mice revealed that although serum and liver iron concentrations were not different between JAK2 mutant mice, ERFE expression was significantly higher and hepcidin expression was significantly lower in *exon 12* mutant mice relative to *V617F* mutant mice [99]. Lastly, erythroblasts from *JAK2 exon 12* mutated PV patients demonstrated increased ERFE expression compared to *JAK2 V617F* PV erythroblasts, suggesting that specific regions in JAK2 may influence iron metabolism by nuanced changes of EpoR signaling [99].

To further explore the regulation of iron metabolism in PV, we compared parameters of iron metabolism (unpublished data) in patients with erythrocytosis who lacked a mutation in JAK2 and those with a JAK2 mutation (i.e., V617F or exon 12) (Table I). Markers of iron metabolism and erythropoiesis were comprehensively evaluated in 22 patients with wild-type and 19 patients with mutant JAK2. JAK2 mutation was evaluated by sequencing at the JAK2 gene locus. Most individuals with wild-type JAK2 had no underlying explanation for erythrocytosis and potential hypoxic causes for erythrocytosis were found in only 4 of 22 patients (1 tracheal cannulation, 1 laryngeal carcinoma, and 2 smokers).

**Table I:** Markers of iron metabolism and erythropoiesis in patients with erythrocytosis

	Age	Hb	MCV	EPO	sTfR1	Fe	Tf sat	Ferritin	Hepcidin	ERFE
Units	Years	g/dL	fL	U/L	nmol/L	ug/mL	%	ng/mL	ng/mL	ng/mL
Normal ranges		13-17	80-96	4.1-19.5	0.9–2.3	65-180	20-50	12-300	29-254	12 ± 9^a^
JAK2 WT	57 ± 13	18 ± 1	90 ± 6	10 ± 4	4 ± 1	24 ± 13	34 ± 20	204 ± 180	78 ± 33	7 ± 4
JAK2 MUT	73 ± 11	19 ± 2	85 ± 8	3 ± 3	8 ± 6	12 ± 6	18 ± 11	70 ± 102	45 ± 35	24 ± 19
*P* value	0.0002	0.22	0.02	< 0.0001	0.005	0.001	0.006	0.01	0.006	0.0003

*Hb* hemoglobin, *MCV* mean corpuscular volume, *EPO* serum erythropoietin, *sTfR1* soluble transferrin receptor 1, *Fe* serum iron, *Tf sat* transferrin saturation, *ERFE* serum erythroferrone, *JAK2 WT* wild type JAK2, *JAK2 MUT* mutant JAK2 (V617F or exon 12)

^a^Adapted from ref. [88]

JAK2 mutated patients exhibit iron-restricted erythropoiesis, with lower MCVs and higher sTfR1; systemic iron deficiency, with decreased serum iron, ferritin, and transferrin saturation; and expected lower serum Epo concentration relative to patients with wild-type JAK2 (Table I). We anticipated and observed increased ERFE levels and consequent hepcidin suppression in JAK2 mutated patients (Table I). Relative suppression of hepcidin would be expected to result in iron mobilization from hepatocyte stores, export after recycling by splenic macrophages, and increased iron absorption by duodenal enterocytes. Such influx of iron into the circulating compartment would be expected to result in recovery from iron deficiency, but JAK2 mutated patients, compared to patients with erythrocytosis and wild-type JAK2, instead exhibit greater iron deficiency (Table I). Hepcidin suppression without recovery from iron deficiency raises several possibilities:

1. The degree of increased ERFE in PV patients is significantly lower relative to that in patients with β-thalassemia in whom erythropoiesis is also expanded but ineffective, resulting in the accumulation of basophilic and polychromatophilic erythroblasts with a higher expression of ERFE [87] (Fig. 4). Without the accumulation of these erythroblasts, ERFE expression is not sufficient to suppress hepcidin to the degree required to enable recovery from iron deficiency.

**Fig. 4:**
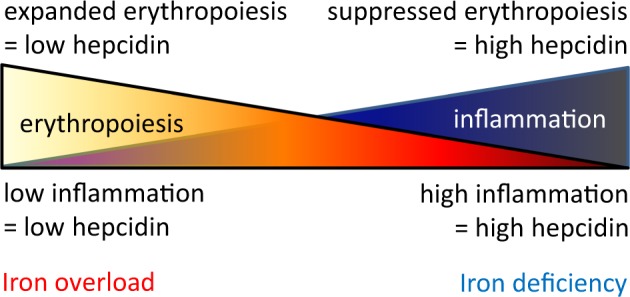
Competitive hepcidin regulation. Hepcidin expression is enhanced by inflammation and suppressed by erythropoiesis such that the combination of both conditions would theoretically lead to equal opposite directional effects on hepcidin regulation in PV, a disease of concurrent expanded erythropoiesis and inflammation
2. Additional factors co-exist to concurrently induce upregulation of hepcidin. Previous reports suggest that hepcidin expression is higher than expected in PV patients [100]. This is in line with the known association between PV and systemic inflammation [101], increasing hepcidin expression by an IL-6-mediated mechanism as in other chronic inflammatory conditions [102] (Fig. 4). Furthermore, activation of JAK/STAT signaling induces IL-6 production [103] with fivefold elevated plasma IL-6 concentration in *JAK2 V617F* mice, decreased to normal levels after treatment with JAK2 inhibitor [104]. Confounding this possibility is the expectation of increased ferritin due to inflammation.
3. Persistent iron deficiency in PV may be a consequence of decreased iron absorption. Evidence of altered iron absorption in PV patients is scarce. A single study evaluating iron absorption in 10 PV patients demonstrates that some degree of impaired iron absorption may be present in a proportion of patients [104]. This is surprising because iron absorption is *negatively* regulated by hepcidin such that hepcidin suppression in PV would suggest *enhanced* iron absorption. However, because of a physiologically hypoxic intestinal lumen and the finding that hypoxia directly influences iron uptake into duodenal enterocytes [95], hypoxia may support iron absorption in a partially hepcidin-independent manner. Thus, if erythrocytosis in PV enables the delivery of more than physiological oxygen to the intestine and thus reduces hypoxia, the loss of normal hypoxic mechanisms in PV may prevent iron uptake into duodenal enterocytes, decreasing available iron for absorption despite hepidin suppression (Fig. 3).
4. Persistent iron deficiency in PV may also be a consequence of frequent gastrointestinal bleeding. Although the underlying mechanisms are unclear, pathological bleeding has been reported in 8.1% of PV patients, most commonly in the gastrointestinal tract [105]. This small fraction of PV patients with clinically significant bleeding may reflect a higher prevalence of minor bleeding that could account for iron deficiency at diagnosis of PV in a larger proportion of patients. Because chronic microscopic bleeding is often implicated in iron deficiency anemia [106], we postulate this also as a possible but less likely cause of systemic iron deficiency in PV patients. Thus, a study of chronic microscopic bleeding, to document occult blood loss in this patient population, would be informative.
5. Persistent systemic iron deficiency may be a consequence of an aberrant iron restriction response in PV patients. Iron restriction is thought to involve the development of erythroblast resistance to Epo, decrease erythroid precursor sensitivity to inflammation in an Epo-independent manner, and selectively enable cell survival without inducing differentiation [31–34]. Thus, if iron restriction normally serves as a brake on erythropoiesis when iron availability is limited, aberrant inflammation-insensitive erythropoiesis in PV may hijack iron for hemoglobin synthesis at the expense of iron requirements for all other cell functions, depleting iron stores.

We anticipate that the cause of iron deficiency in PV is a composite of multiple concurrent factors, most likely an aberrant iron restriction response in conjunction with altered hypoxia regulation of iron absorption.

## Potential therapeutic applications

Several novel ways to treat PV patients may arise from understanding concurrent regulation of hepcidin by iron status, inflammation, and erythropoiesis in PV. Studies in *JAK2 V617F* mice demonstrate the potential of exogenous hepcidin to reverse erythrocytosis, decrease splenomegaly, and sequester iron in splenic macrophages [98], and suggest that the use of such “hepcidin mimetic agents” may be beneficial in low-risk PV patients. Several strategies are being used to induce endogenous hepcidin production or to exogenously mimic its activity. Hepcidin mimetics, including full-length exogenous hepcidin or truncated forms called “mini-hepcidin,” stimulators of endogenous hepcidin production, ERFE antagonists, and ferroportin blockers, are all in various stages of clinical development [107]. These agents are expected to induce iron-restricted erythropoiesis without contributing to systemic iron deficiency. Hepcidin therapy thus has the potential to accomplish the equivalent of “chemical therapeutic phlebotomy” and might reduce the need for cytoreductive therapy in low-risk PV patients with symptomatic iron deficiency and/or high phlebotomy requirements. Furthermore, increasing hepcidin in PV may improve PV-associated symptoms as a consequence of hepcidin’s anti-inflammatory function [108]. Taken together, specifically targeting iron availability for erythropoiesis, by sequestering iron in macrophages and preventing further iron absorption, may circumvent the systemic symptoms of iron deficiency while restricting iron availability needed for accelerated erythropoiesis in PV. The consequence of such therapy would potentially obviate the need for therapeutic phlebotomy in low-risk PV patients, reversing iron deficiency in those who present with or develop signs and symptoms of iron deficiency during their treatment course, and prevent early administration of cytoreductive agents to otherwise low-risk PV patients.

Of note, pathways involved in iron metabolism are attractive potential targets for therapy in multiple conditions of iron overload and anemia [107, 109] such as transfusion-dependent myelofibrosis. In this scenario, hepcidin mimetics are expected to redistribute iron from parenchymal cells (i.e., liver, heart, and pancreas) into macrophages, cells specifically designed for handling iron released from senescent RBCs during erythrophagocytosis. While this re-distribution of iron is *paradoxically* beneficial for iron-loaded anemias, we anticipate that it is even more physiologically relevant in PV where sequestration of iron in macrophages prevents its utilization in hemoglobin synthesis.

## Conclusion

Patients with PV present with iron deficiency despite expanded erythropoiesis and recurrent therapeutic phlebotomy induces further degrees of iron deficiency. Significant progress has been made in defining the molecular basis of PV as well as physiological and pathological regulation of iron metabolism. Furthermore, together with newly delineated molecular mechanisms involved in the iron restriction response, this knowledge enables a more nuanced evaluation of aberrant erythroid regulation of iron metabolism and iron sensing which regulate erythropoiesis in PV. Understanding the dysregulation of iron metabolism in PV may enable the development of novel therapeutics. In the interim, exploring chemical means of inducing erythropoiesis-targeted iron restriction may benefit PV patients requiring repeat therapeutic phlebotomy.
